# Editorial: Cardiac fibroblasts: from development to disease

**DOI:** 10.3389/fcell.2025.1736508

**Published:** 2025-12-02

**Authors:** Silvia C. Hernandez, Thomas Moore-Morris, Adrian Ruiz-Villalba

**Affiliations:** 1 The Wilf Family Cardiovascular Research Institute, Department of Medicine (Cardiology), Albert Einstein College of Medicine, Bronx, NY, United States; 2 Department of Microbiology and Immunology, Albert Einstein College of Medicine, Bronx, NY, United States; 3 IGF, Univ Montpellier, CNRS, Inserm, Montpellier, France; 4 Department of Animal Biology, Faculty of Sciences, University of Málaga, Campus de Teatinos s/n, Málaga, Spain; 5 Instituto de Investigación Biomédica de Málaga y Plataforma en Nanomedicina (IBIMA Plataforma BIONAND), Málaga, Spain

**Keywords:** cardiac fibroblasts, extracellular matrix, fibrosis, neutrophils, development, cardiac disease

Cardiac fibroblasts (CFs) have been a major focus of study in recent years in the field of cardiovascular research. They are the main cell-type responsible for producing and remodelling the extracellular matrix (ECM), a dynamic scaffold that supports cardiac cells during development, in health and upon cardiac injury (Cadosch et al., 2024). Thanks to the recent advances in high-throughput technologies, CFs are now recognized as a heterogeneous group of cells with diverse transcriptomic, developmental, and functional characteristics. However, our understanding of their functional heterogeneity, developmental origins, and interactions with other key components–such inflammatory cells and the extracellular matrix—remains incomplete in all these contexts. This Research Topic, *Cardiac Fibroblasts: From Development to Disease*, presents four novel contributions that address some of these open questions related to CF biology.

In their article, Harrington and Moore-Morris. provide a comprehensive review of current challenges associated with the specificity of CF markers in different contexts, resulting from the use of diverse genetic tools, antibodies for identification, and lineage tracing approaches in mice and zebrafish. These different approaches and models indicate that the embryonic epicardium is the main source of CFs during development, and that fibrosis primarily results from the activation of these cells, with minor contributions from other sources. In addition, the authors highlight the role of CFs in fibrosis across different heart failure models, such as myocardial infarction (MI) and transverse aortic constriction (TAC), and in regenerative models, such as zebrafish and neonatal mice, stressing the main differences between the two scenarios. Interestingly, the molecular pathways that regulate this fibrotic response are conserved across models: transforming growth factor-β (TGF-β), the renin-angiotensin-aldosterone system (RAAS), tumour necrosis factor-α (TNFα), Wingless/Int (Wnt), and more recently studied mediators such as endothelin-1, galectin-3, and interleukin 11, all play a role in the transition of homeostatic CFs into more responsive “activated CFs”, following injury.

Another universal feature of fibrosis is that it is exacerbated during old age. Hence, with the increasing pace of population ageing, understanding the mechanisms involved is highly significant, and it is essential to design cardiac remodeling studies aimed at developing targeted therapies. For this reason, in their original article, Perreault et al. decided to examine the effects of foetal and adult ECM on fibroblast phenotypes. Their initial findings indicate that the developmental age of the ECM influences CF activation and migration rate and stiffness. Notably, CFs cultured on adult compared to fetal ECM expressed higher levels of myofibroblast marker αSMA. These differences were attenuated by stimulation with TGF-β1, suggesting that ECM age-related effects on CF biology were mediated by changes in TGF-β signalling. In line with this, altered TGF-β signalling has been linked to ageing (Ren et al., 2023), and the ECM plays a key role in determining the distribution and activity of TGFβs and other signalling molecules (Alshoubaki et al., 2023).

One of the most important factors in CF activation is inflammation. Although many studies have focused on macrophage-CF crosstalk (Frangogiannis, 2025), interactions of CFs with other prominent immune cell types have been less explored. Recently, neutrophils have been shown to contribute to fibrosis by modulating fibroblast function, promoting healing and scar formation, and depositing fibrotic matrix in wounds (Fischer et al., 2022). In their original research, Anfossi et al. investigated CF-neutrophil interaction, a relationship that is still poorly understood, using primary rat CFs. Here, applying basic cellular and molecular *in vitro* techniques, they demonstrated that CFs are more responsive to interactions with neutrophils under inflammatory conditions compared to basal conditions. Notably, this interaction had a significant effect on the ECM due to the modulation of Matrix Metalloproteinase (MMP) activity by neutrophils in a paracrine manner. Interestingly, the authors concluded that the effects of CF-neutrophil interactions are counteracted by the interferon-beta (IFN-β)/Janus kinases (JAK) IFN-β/JAK signalling pathway.

The molecular mechanisms downstream of the multiple external cues that promote fibroblast activation, from quiescent to pro-fibrotic “myofibroblast” state, are of high clinical interest. Acharya et al. developed an innovative perspective on how RNA-binding motif (RBM) proteins-dependent post-transcriptional gene regulation contributes to this switch during pathological cardiac remodeling. Their review highlights the importance of alternative splicing regulation in CF biology mediated by different classes of RBM proteins such as RNA binding Fox-1 homologs (Rbfox), Muscleblind-like 1 (MBNL1), CUG-BP and ETR-3-like factors (CELF), and serine/arginine-rich splicing factor (SRSF) proteins. Among them, MBNL1 is suggested as a master regulator of the transformation of CFs into their activated state. They also highlighted the role of other RBM proteins in regulating the balance between RNA stability and degradation and indicated that proteins such as Human antigen R (HuR) also contribute to the transition of CFs into a more fibrotic phenotype.

In summary, recent studies, including those featured in this special issue, have enabled significant progress to be made on defining CF origins, markers, heterogeneity, and interactions with other cell types or ECM. However, shedding light on CF diversity has brought new challenges to the field. These include reaching a consensus on the characteristics that define CF popuations in developmental and pathological contexts. Furthermore, as for other interstitial cell types present throughout the body, current approaches for targeting CFs *in vivo* for functional studies, e.g., conditional deletion or AAV-mediated delivery, lack specificity and efficiency. Addressing these hurdles will be critical for identifying novel targets to regulate CF activity for clinical applications. We extend our sincere gratitude to all the authors and reviewers for their invaluable contributions. We hope this Research Topic will inspire further collaborations and advance our understanding of CF biology, ultimately accelerating the development of potential therapeutic targets and personalized medicine.
